# Clinicopathological Significance and Prognostic Values of Long Noncoding RNA *BCYRN1* in Cancer Patients: A Meta-Analysis and Bioinformatics Analysis

**DOI:** 10.1155/2022/8903265

**Published:** 2022-07-14

**Authors:** Xiaoyong Han, Yongfeng Wang, Rangyin Zhao, Guangming Zhang, Chenhui Qin, Liangyin Fu, Haojie Jin, Xianglai Jiang, Kehu Yang, Hui Cai

**Affiliations:** ^1^Graduate School, Ning Xia Medical University, Yinchuan 750004, Ning Xia, China; ^2^General Surgery Clinical Medical Center, Gansu Provincial Hospital, Lanzhou 730000, Gansu, China; ^3^Key Laboratory of Molecular Diagnostics and Precision Medicine for Surgical Oncology in Gansu Province, Gansu Provincial Hospital, Lanzhou 730000, Gansu, China; ^4^Gansu University of Chinese Medicine, First Clinical Medical College, Lanzhou 730000, Gansu, China; ^5^The First Clinical Medical College of Lanzhou University, Lanzhou 730000, Gansu, China; ^6^Evidence-Based Medicine Center, School of Basic Medical Sciences, Lanzhou University, Lanzhou 730000, China; ^7^NHC Key Laboratory of Diagnosis and Therapy of Gastrointestinal Tumor, Gansu Provincial Hospital, Lanzhou 730000, China

## Abstract

**Background:**

Although combination therapies have substantially improved the clinical outcomes of cancer patients, the prognosis and early diagnosis remain unsatisfactory. As a result, it is critical to look for novel indicators linked to cancer. Despite a number of recent studies indicating that the lncRNA brain cytoplasmic RNA1(*BCYRN1*) may be a potential predictive biomarker in cancer patients, *BCYRN1*'s prognostic value is still being debated.

**Methods:**

We utilized PubMed, Embase, Web of Science, and the Cochrane Library to search for studies related to *BCYRN1* until October 2021. Valid data were extracted after determining the articles according to the inclusion and exclusion criteria, and forest plots were made using Stata software. We used hazard ratios (HRs) or odds ratios (ORs) with 95% confidence intervals to evaluate the relationship between abnormal *BCYRN1* expression and patient prognosis and clinicopathological characteristics.

**Results:**

Meta-analysis revealed that increased *BCYRN1* expression was associated with both overall tumor survival (OS; HR = 1.84, 95% CI 1.51–2.25, *p* < 0.0001) and disease-free survival (DFS; HR = 1.65, 95% CI 1.20–2.26, *p*=0.002). Furthermore, a strong association was discovered between increased *BCYRN1* expression and tumor invasion depth (OR = 2.11, 95% CI 1.49–2.99, *p*=0.000), clinical stage (OR = 2.52, 95% CI 1.18–5.37, *p*=0.017), and distant tumor metastasis (OR = 4.19, 95% CI 1.45–12.05, *p*=0.008).

**Conclusions:**

We found that high *BCYRN1* expression was associated with poor survival prognosis and aggressive clinicopathological characteristics in various cancers, indicating that it is a potential prognostic indicator as well as a therapeutic target. Further research is needed on pan-cancer cohorts to determine the clinical relevance of *BCYRN1* in distinct cancer types.

## 1. Introduction

Since the turn of the new century, cancer incidence and mortality have gradually exceeded that of other chronic diseases [1]. According to the most recent *CA*, a cancer journal, estimates, 1.9 million new cases of cancer were diagnosed in the United States in 2021, with an estimated 610,000 deaths [2]. Although combination therapies, such as surgery, chemotherapy, radiation therapy, targeted therapy, and immunotherapy, have substantially improved the clinical outcomes of cancer patients, the prognosis and early diagnosis remain unsatisfactory [3]. In recent years, advances in screening techniques, targeted therapies, immunotherapies, bioinformatics, and cancer biology have identified novel biomarkers for early diagnosis and prognosis prediction [4], and novel tumor marker detection techniques are important [5–8].

Long non-coding RNAs (lncRNAs) have gained considerable interest as cancer biomarkers in recent years with the advent of next-generation sequencing technologies [9]. The lncRNAs have a length of more than 200 nucleotides and lack any protein-coding activity [10]. They regulate gene expression at the transcriptional level through chromatin remodeling and miRNA sponging, at the post-transcriptional level by affecting RNA splicing and stability and at the translational level by controlling signal transmission [11, 12]. Studies increasingly show that aberrant lncRNA expression is linked to biological processes such as tumor growth, angiogenesis, metastasis, and invasion, and lncRNAs can be exploited as tumor suppressor genes or oncogenes for cancer therapy and prevention [12, 13].

The lncRNA brain cytoplasmic 200 (BC200), also known as brain cytoplasmic RNA1 (*BCYRN1*), is normally expressed in neurons and is implicated in cancer and neurological diseases [14]. Studies show that *BCYRN1* is overexpressed in non-small-cell lung cancer [15–17], hepatocellular carcinoma [18–20], colorectal cancer [21–24], bladder cancer [25, 26], esophageal squamous cell carcinoma [27, 28], gastric cancer [29, 30], cervical cancer [31], ovarian cancer [32], and breast cancer [33] tissues compared to matched normal tissues. However, no systematic review has been conducted so far on the pan-cancer data of *BCYRN1*. To this end, we performed a meta-analysis of the relevant studies to further evaluate whether *BCYRN1* is a reliable prognostic biomarker and therapeutic target for different cancers by evaluating the correlation between *BCYRN1* expression levels and cancer-related clinicopathological features and patient prognosis. Finally, the clinicopathological and prognostic value of *BCYRN1* in cancer patients was validated by bioinformatics analysis of cancer databases.

## 2. Materials and Methods

### 2.1. Search Strategy for Literature

All procedures mentioned below were performed in accordance with PRISMA Checklist protocols [34]. Prior to October 1, 2021, PubMed, Web of Science, Embase, and the Cochrane Library were used to search for relevant papers studying the association between lncRNA *BCYRN1* expression and clinical outcomes in cancer patients. Medical Subject Headings (MESH) keywords and free terms were merged in this search. Our search keywords are as follows: (“LncRNA” OR “Long non-coding RNA”) AND (“BCYRN1” OR “BC200” OR “BC200a” OR “LINC00004” OR “NCRNA00004” OR “Brain cytoplasmic RNA1”) AND (“Neoplasms” OR “Carcinoma” OR “Tumor” OR “Cancer”). To guarantee accuracy and consistency, two writers independently assessed the database search approach and discussed the results.

### 2.2. Inclusion and Exclusion Criteria

The duplicate articles were first eliminated, and the titles and abstracts of the remaining studies were screened on the basis of the following inclusion criteria: (1) patients with histopathologically proven cancer; (2) analysis of cancer tissues and adjacent normal tissues; (3) detection of *BCYRN1* levels by qRT-PCR; (4) the paper included clinical factors such as age, gender, tumor size, TNM stage, clinical stage, lymph node metastasis, or distant metastases, as well as prognostic markers such as overall survival (OS), disease-free survival (DFS), or progression-free survival (PFS); (5) demarcation of patients into *BCYRN1* low and *BCYRN1* high expression groups based on the cut-off value, with the number of patients in each group explicitly specified; (6) survival hazard ratios (HRs) and 95% confidence interval (CI) by multivariate analysis or Kaplan–Meier (K-M) curves; and (7) published in the English language. Exclusion criteria are as follows: (1) studies describing other lncRNAs or lncRNAs unrelated to cancer; (2) duplicate articles; (3) other types of literature, such as reviews, letters, conference abstracts, meta-analyses, case reports, and so on; (4) articles focusing on biological functions and related mechanisms; and (5) a lack of sufficient HR and 95% CI to extract data.

### 2.3. Data Extraction and Quality Evaluation

The following information should be extracted from eligible literature: first author, publication year, country, tumor type, sample type, sample size (high/low), cutoff of *BCYRN1* expression, analysis method, survival (OS/RFS/PFS), HR availability, HR (95%CI) with *p* value, month of follow-up, and Newcastle–Ottawa Scale score (NOS). Survival HRs (95% CI) were retrieved indirectly from K-M curves using the Engauge Digitizer tool in case multivariate analysis had not been performed. The NOS scoring criteria (scores from 0 to 9) were used to assess the quality of the included studies, and those with scores >6 were included in the meta-analysis.

### 2.4. Statistical Analysis

We used log HR and SE to summarize survival outcomes, while OR and corresponding 95% CI were applied to summarize clinicopathological parameters. In addition, between-study heterogeneity was assessed by the *x*^2^ test and *I*^2^ statistic. *Q* test (*P*_*Q*_) *p* value < 0.05 and *I*^2^ > 50% indicated that there was statistical heterogeneity among studies, and a random-effects model was used to analyze the results. In other cases, a fixed-effects model was employed. We used forest plots to present the meta-analysis results and used the Begg test to assess any prospective bias in the publications. Sensitivity analyses were performed by sequentially removing individual included studies to test whether the overall pooled estimate was stable. Analyses were performed using Stata 12.0 for Windows (Stata, College Station, TX, USA), and *p* < 0.05 was considered statistically significant.

### 2.5. To Identify the Differential Expression of *BCYRN1* Gene in Human Cancers

UCSC Xena (https://xena.ucsc.edu/ originated from TCGA database) was used to retrieve RNA sequences, somatic mutations (SNPs and short INDELs), clinicopathologic, and survival data for 33 malignancies. We picked the ONCOMINE database (http://www.oncomine.org/) to acquire a complete knowledge of *BCYRN1* expression in pan-cancers utilizing many data sets. As a result, the levels of *BCYRN1* in various cancer types were assessed after a particular threshold was set (*p* value = 0.05, fold change = 1.5). Perl software was used to extract and combine *BCYRN1* expression levels for TCGA pan-cancer analysis. The “Wilcoxon test” method was used to investigate the differential expression of *BCYRN1* in various cancer types. As a threshold, a false discovery rate (FDR) of 0.05 was chosen. “^*∗*^”, “^*∗∗*^”, and “^∗∗∗^” represent FDR <0.05, <0.01, and <0.001, respectively. Box plots were then created with the R-package “ggpubr.” The cBioPortal database (https://www.cbioportal.org/) was used to assess changes in *BCYRN1* expression in various cancer types [35].

### 2.6. Association between *BCYRN1* Expression and TMB or MSI in Pan-Cancer

Tumor mutational burden (TMB) was computed using the Perl script and divided by the entire length of the exons to count the number of mutations in each tumor sample (i.e., 33 tumors using somatic mutation data and corrected to a number of mutated bases per 1 million bases). The microsatellite instability (MSI) score came from the TCGA website. The “cor. test” command was used to do Spearman's method correlation study between cancer gene expression and TMB or MSI. A radar map was created using the R-package “fmsb” to view both indications.

### 2.7. Verification of Survival Outcomes in the GEPIA Database

Gene expression profiling interaction analysis (GEPIA) was performed according to the Cancer Genome Atlas (TCGA) data set to further validate the prognostic relevance of *BCYRN1* overexpression in tumor tissues. TCGA and GTEx data were matched in various tumors, with a cutoff of *p* < 0.01. OS and DFS of *BCYRN1* in pan-cancer were plotted using the Kaplan–Meier method.

## 3. Results

### 3.1. Screening Process for Eligible Literatures

**Figure 1:**
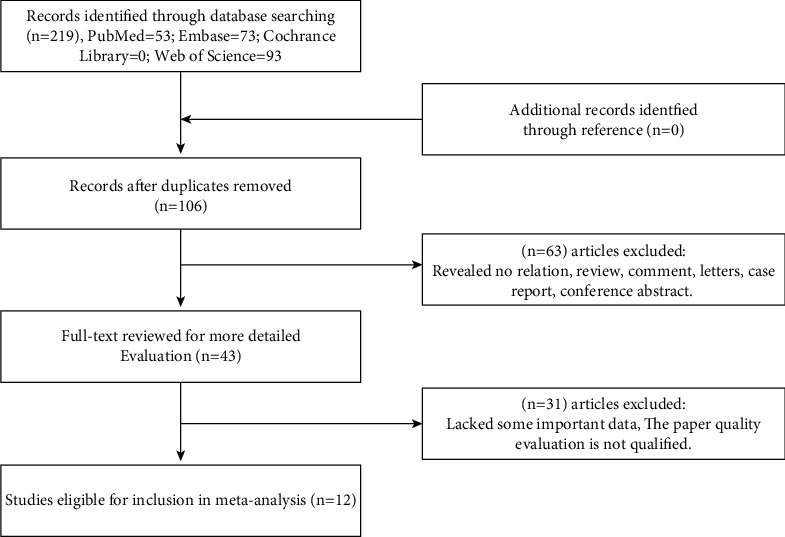
Flow diagram of this meta-analysis.

The cancer-related gene *BCYRN1* was thoroughly searched in four major English databases: PubMed (*n* = 53), Web of Science (*n* = 93), Embase (*n* = 73), and Cochrane Library (*n* = 0). After deleting duplicates (*n* = 106), the remaining papers' titles and abstracts (*n* = 113) were examined and appraised. Sixty-three articles were rejected owing to the aims, article type (reviews, case studies, or conference abstracts), or unrelated findings. Forty-three full-text articles were downloaded, of which 31 were rejected following preliminary analysis due to lack of significant data or unsatisfactory quality of the data. Finally, 12 studies with sufficient data on survival and clinical features were included in the meta-analysis. The procedure is outlined in Figure 1.

### 3.2. Characteristics of Included Research Projects

All included studies had been conducted in China and comprised 1,284 patients. The articles were published between 2016 and 2021. Two studies looked into hepatocellular carcinoma; two looked into colorectal cancer; and the remaining studies looked into bladder cancer, extranodal NK/T-cell lymphoma, glioblastoma, gastric cancer, prostate cancer, colon cancer, cancer, and esophageal squamous cell carcinoma. *BCYRN1* expression in cancer and para-cancer tissues was detected by qRT-PCR. The patients were demarcated into the *BCYRN1* low and *BCYRN1* high groups, and the cutoff was the median expression level in five studies and mean expression in three studies. No cutoff value was indicated in the remaining four studies. Only two studies included the DFS and one PFS, whereas 8 studies provided OS. The HR and 95% CI of three studies were obtained directly from the multivariate regression analysis, and that for the remaining six were extracted from the K-M survival curves using Engauge Digitizer software. The duration of follow-up ranged from 40 to 96 months. The NOS scores of the studies were 6 to 8. The data are summarized in Table 1.

### 3.3. Association of *BCYRN1* Level with Survival Outcome

**Figure 2:**
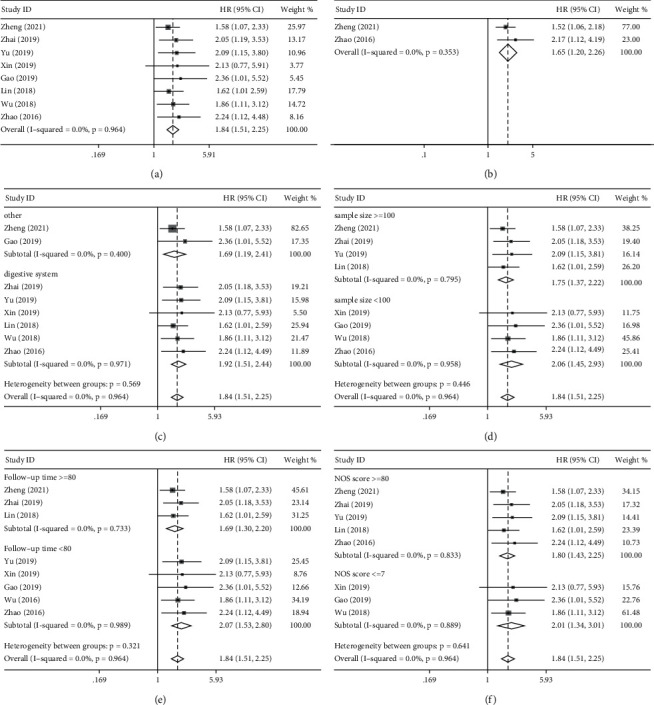
Forest plots for the association of *BCYRN1* expression with overall survival, disease-free survival, and subgroup analysis of *BCYRN1* expression with overall survival: (a) forest plots for the association of *BCYRN1* expression with overall survival, (b) forest plots for the association of *BCYRN1* expression with disease-free survival, (c) subgroup analysis stratified by type of cancer, (d) subgroup analysis stratified by sample size, (e) subgroup analysis stratified by follow-up time, and (f) subgroup analysis stratified by NOS score.

Eight studies including 1,028 cancer patients investigated the link between *BCYRN1* levels and OS. Since no significant heterogeneity was found (*I*^2^ = 0.0%, *p*=0.964), we performed a pooled analysis using a fixed-effect model. Pooled HRs indicated that high *BCYRN1* levels were strongly associated with worse OS (HR = 1.84, 95% CI 1.51–2.25, *p* < 0.001; Figure 2(a)). In addition, only two studies (280 patients) were included to assess the association of *BCYRN1* expression with DFS. Consistent with the OS results, increased *BCYRN1* expression was found to be associated with unfavorable DFS (HR = 1.65, 95% CI 1.20–2.26, *p*=0.002; Figure 2(b)). Furthermore, we conducted subgroup analyses to look into the relationship between *BCYRN1* expression levels and OS based on the cancer type (digestive or other systems; Figure 2(c)), sample size (≥100 or <100 tissues; Figure 2(d)), follow-up time (≥80 or <80 months; Figure 2(e)), and article quality (NOS score ≥8 or≤7; Figure 2(f)). There was no evidence of considerable heterogeneity within groups, and the findings of the subgroup analysis had no effect on *BCYRN1*'s ability to predict OS in these malignancies.

### 3.4. Association of *BCYRN1* Expression with Clinicopathologic Parameters

**Figure 3:**
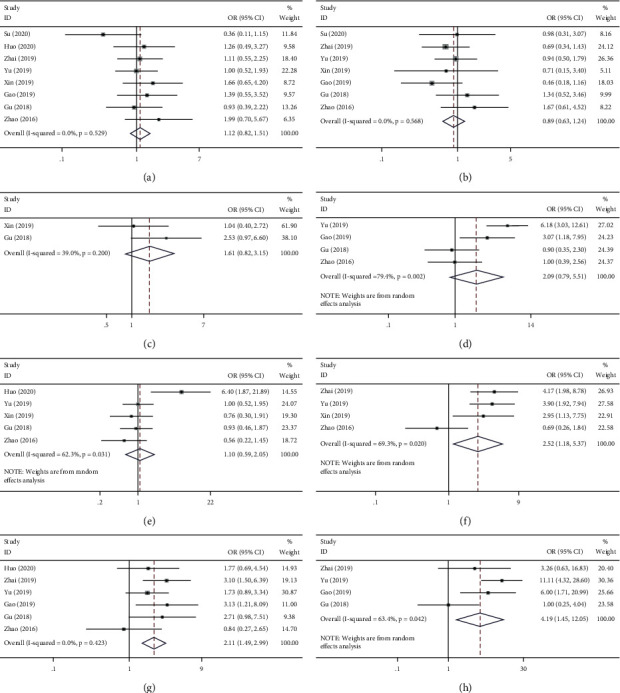
Forest plots for the association of *BCYRN1* expression with clinicopathological features: (a) age, (b) gender, (c) TNM stage, (d) lymph node metastasis, (e) distant metastasis, (f) tumor T stage, (g) tumor size, and (h) differentiation grade.

The results showed that overexpression of *BCYRN1* was associated with age (≥60 vs.<60, OR = 1.12, 95% CI 0.82–1.15, *p*=0.475; Figure 3(a)), gender (male vs. female, OR = 0.89, 95% CI 0.63–1.24, *p*=0.568; Figure 3(b)), tumor size (large vs. small, OR = 1.61, 95% CI 0.82–3.15, *p*=0.166; Figure 3(c)), lymph node metastasis (positive vs. negative, OR = 2.09, 95% CI 0.79–5.51, *p*=0.135; Figure 3(d)), and tumor differentiation (poor vs. good, OR = 1.10, 95% CI 0.59–2.05, *p*=0.774; Figure 3(e)) that were not significantly associated, and the results were not found to be statistically significant. However, high expression of *BCYRN1* was observed to be significantly associated with some advanced clinical features, including TNM stage (III-IV vs. I-II, OR = 2.52, 95% CI 1.18–5.37, *p*=0.017; Figure 3(f)), T stage of the tumor (III-IV vs. I-II, OR = 2.11, 95% CI 1.49–2.99, *p*=0.000; Figure 3(g)), and tumor distant metastasis (positive vs. negative, OR = 4.19, 95% CI 1.45–12.05, *p*=0.008; Figure 3(h)). A fixed-effects model was used for low heterogeneity (0–50%), while a random-effects model was used for large heterogeneity (>50%). Data pertaining to the forest plot of survival prognosis and clinical pathology are recorded in Table 2.

### 3.5. Publication Bias and Sensitivity Analysis

**Figure 4:**
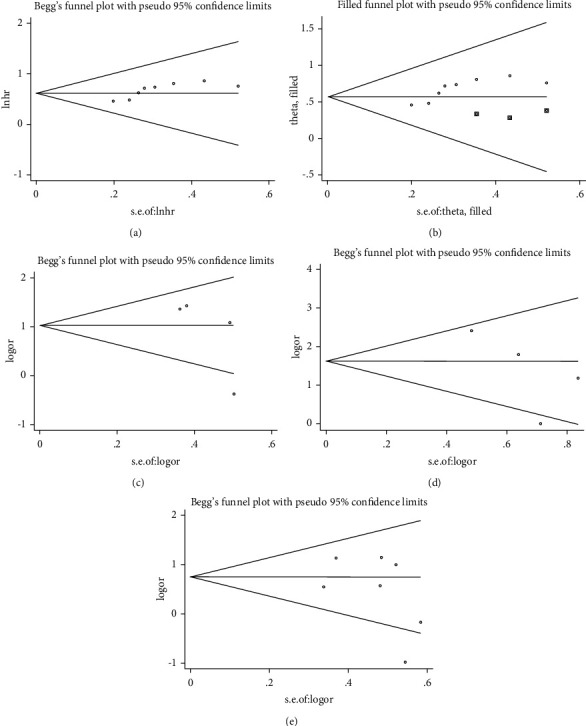
Begg's publication bias plots: (a) OS, (b) OS after clipping, (c) TNM stage, (d) distant metastasis, and (e) tumor T stage.

**Figure 5:**
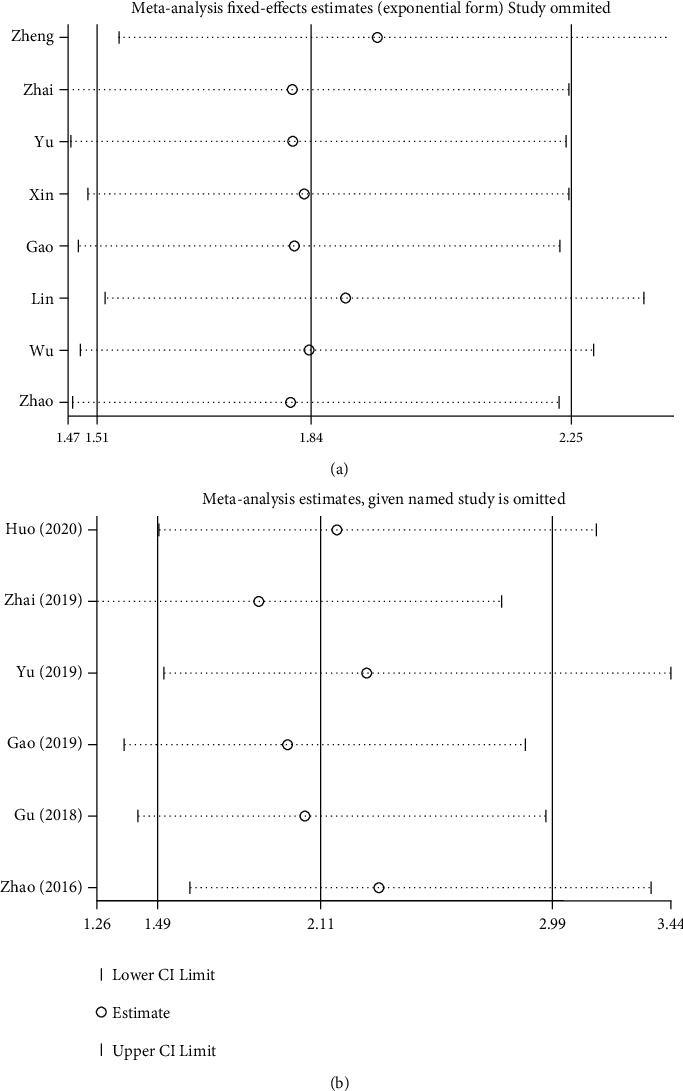
Sensitivity analysis for studies about OS and tumor T stage by omitting each study sequentially: (a) OS and (b) tumor T stage.

Begg's test was used to analyze potential publication bias. For OS, the funnel plot appeared asymmetric, and the Begg test (*p* > |*t*| = 0.019; Figure 4(a)) indicated some publication bias. Using the scissors method, after filling out three imaginary unpublished papers, the funnel plot became symmetrical, and the pooled HR and 95% CI remained stable (HR = 1.768, 95% CI 1.473–2.123, *p* < 0.001; Figure 4(b)) [39]. For pathological parameters with significant differences in pooled ORs, Begg plot data showed TNM stage (*p* > |*t* | = 0.231; Figure 4(c)), distant metastasis (*p* > |*t*| = 0.237; Figure 4(d)), and tumor T stage (*p* > |*t*| = 0.605; Figure 4(e)), indicating no significant publication bias. A sensitivity analysis was performed for OS (Figure 5(a)) and tumor T stage (Figure 5(b)), and the pooled HR and OR changed within a limited range without significant change after deletion of each study, indicating that our results were stable. From this, it can be seen that the relevant conclusions we draw are stable and reliable.

### 3.6. Expression of *BCYRN1* in Pan-Cancers

**Figure 6:**
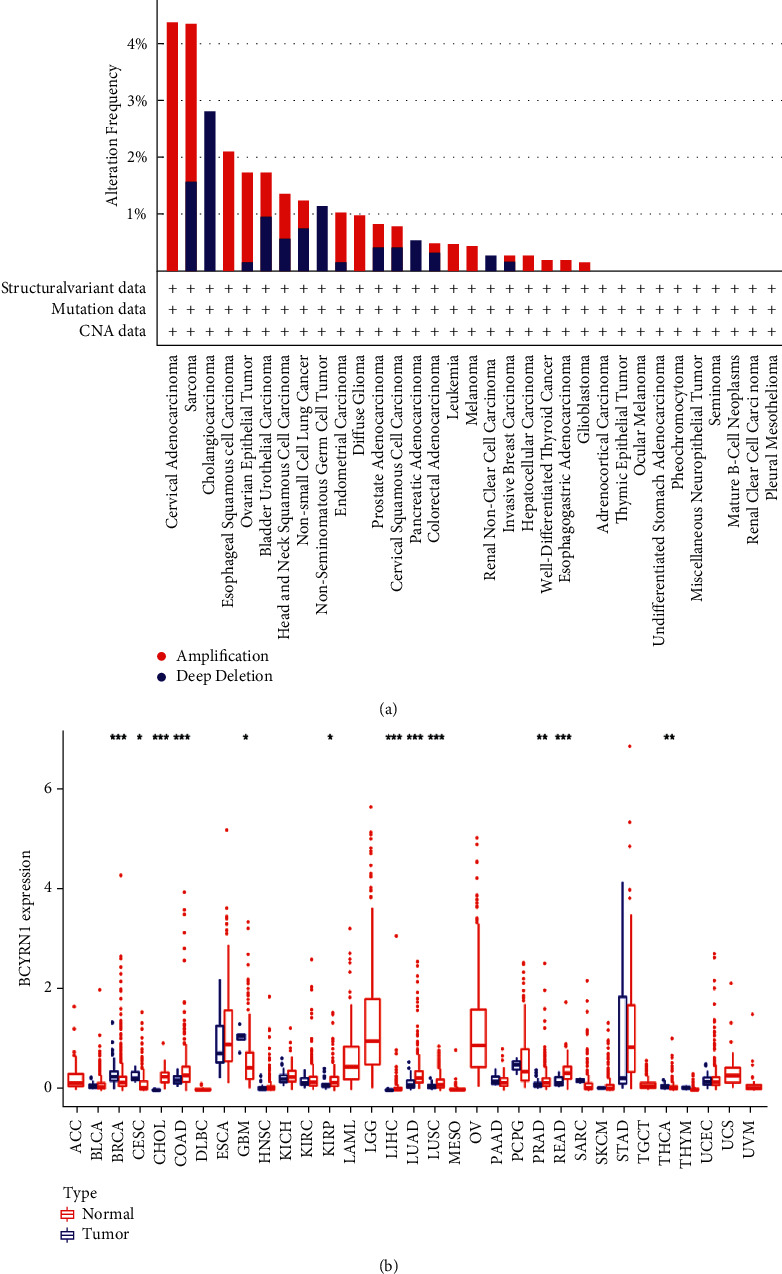
*BCYRN1* gene expression levels and alteration in different cancer types from TCGA: (a) the alteration frequency of the *BCYRN1* gene in different cancers obtained from the cBioPortal and (b) *BCYRN1* gene expression levels in different cancer types from TCGA data. The red fusiformis represents tumor tissue, and the blue fusiformis represents normal tissue. ^*∗*^FDR < 0.05, ^∗∗^FDR < 0.01, and ^*∗∗∗*^FDR < 0.001.

We used R software to examine RNA sequencing data in the TCGA database to further investigate the differential expression of *BCYRN1* in pan-cancers. According to our findings, *BCYRN1* is significantly expressed in multiple cancer types, including CHOL, COAD, KIRP, LIHC, LUAD, LUSC, PRAD, and READ. However, low *BCYRN1* expression was observed in BRCA, CESC, GBM, and THCA (Figure 6(a)). Using the cBioPortal database, we observed the variation of *BCYRN1* in various types of cancer. The correlation results showed that the variation was mainly significant amplification, followed by deep deletion. Among all malignancies, cervical adenocarcinoma had the highest frequency of variants, followed by sarcoma (Figure 6(b)).

### 3.7. Association of *BCYRN1* Expression with TMB and MSI in Pan-Cancer

**Figure 7:**
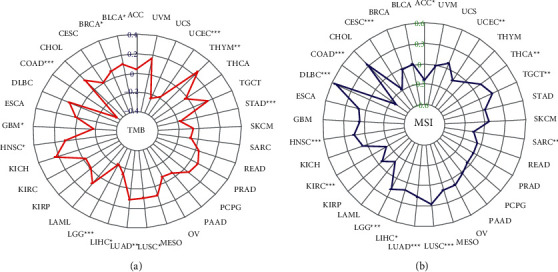
Relationships between *BCYRN1* gene expression and TMB and MSI in pan-cancer. (a) The radar chart illustrated the association between TMB and *BCYRN1* gene expression in different cancers. The red curve represents the correlation coefficient, and the blue value represents the range. (b) The radar chart illustrated the relationship between MSI and *BCYRN1* gene expression in different cancers. The blue curve represents the correlation coefficient, and the green value represents the range. TMB, tumor mutational burden; MSI, microsatellite instability; and ^*∗*^*p* < 0.05, ^*∗∗*^*p* < 0.01, and^*∗∗∗*^*p* < 0.001.

High TMB is a newly identified class of biomarkers related to sensitivity to immune checkpoint inhibitors, including PD-1/PD-L1 inhibition, which can assess the efficacy of immunotherapy in cancer patients [40, 41]. Therefore, it is interesting to investigate the relationship between TMB and *BCYRN1* expression in different types of cancer. The results indicate that *BCYRN1* expression correlates with TMB in a significant number of cancers. *BCYRN1* expression was positively correlated with TMB in six cancer types, including BLCA, BRCA, HNSC, LUAD, LUSC, and THYM. In contrast, *BCYRN1* expression was inversely correlated with TMB in six other cancer types, which included COAD, GBM, LGG, LIHC, STAD, and UCEC (Figure 7(a)).

Recently, it has been found that MSI can be detected in numerous tumors (such as colorectal cancer) and has the potential to be a marker of PD-1 blockade [42, 43]. Therefore, further verification of whether *BCYRN1* expression is associated with MSI in different types of cancer is warranted. The results showed that *BCYRN1* expression was significantly correlated with MSI in 14 cancer types. *BCYRN1* expression was positively correlated with MSI in 8 of the cancer types (DLBC, HNSC, LGG, LIHC, LUAD, LUSC, TGCT, and THCA). In addition, *BCYRN1* expression was inversely correlated with MSI in six other cancer types (ACC, CESC, COAD, KIRC, SARC, and UCEC; Figure 7(b)).

### 3.8. Correlation Analysis between *BCYRN1* Expression and TNM Staging of Pan-Cancer

**Figure 8:**
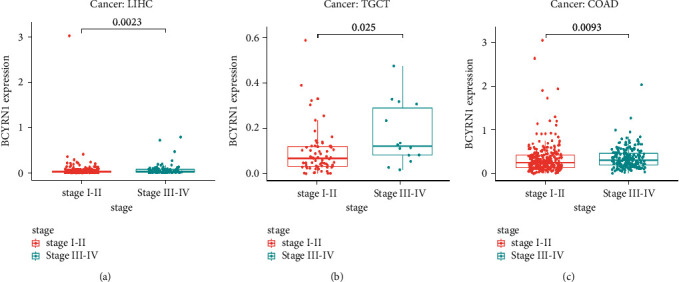
Relationship between *BCYRN1* gene expression and tumor stage in pan-cancer: (a) LIHC, (b) TGCT, and (c) COAD. LIHC, liver hepatocellular carcinoma; TGCT, testicular germ cell tumors; and COAD, colon adenocarcinoma.

*BCYRN1* expression was associated with the clinical stage in several cancers (Figure 8). For LIHC (*p*=0.0023; Figure 8(a)), TGCT (*p*=0.025; Figure 8(b)), and COAD (*p*=0.0093; Figure 8(c)), *BCYRN1* was highly expressed in stage III-IV, but lowly expressed in stage I-II. From this, it can be seen that in the above cancers, high expression of *BCYRN1* is associated with clinical stage progression of cancer and has the potential to be a predictor of tumor prognosis and progression.

### 3.9. Verification of Survival Outcomes in the GEPIA Database

**Figure 9:**
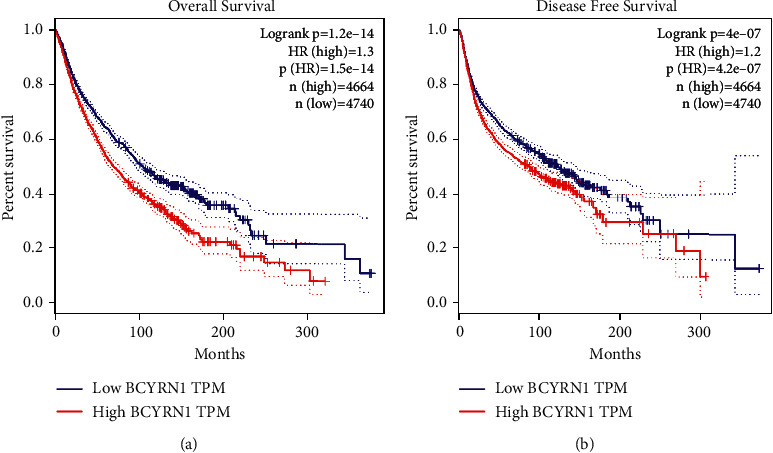
The relationship between *BCYRN1* expression and cancer patient prognosis in the GEPIA cohort: (a) OS plots based on *BCYRN1* expression in 33 types of cancer (*n* (low) = 4,740 vs. *n* (high) = 4,664) and (b) DFS plots based on *BCYRN1* expression in 33 types of cancer (*n* (low) = 4,740 vs *n* (high) = 4,664).

Regarding the relationship between *BCYRN1* expression and prognosis, in the GEPIA cohort, 33 malignancies in 4,740 patients were divided into high and low expression groups according to the median value, and the survival curve showed that upregulation of *BCYRN1* expression was associated with deterioration of OS ((HR = 1.3, log rank *p* < 0.05)) and DFS (HR = 1.2, log rank *p* < 0.05; Figure 9), which confirmed the results of our meta-analysis. These results support our conclusion and suggest that *BCYRN1* may become a novel prognostic biomarker in multiple cancers.

## 4. Discussion

Given the steady increase in the annual rates of cancer incidence and mortality throughout the world, it is estimated that cancer will overtake chronic diseases as the primary cause of death and a major impediment to increasing life expectancy [44]. Despite the recent advances in cancer therapies, most cancer patients have a poor prognosis. Therefore, early diagnosis and treatment are critical to improving patient prognosis. However, the biomarkers currently used in clinical practice lack sensitivity and specificity, thereby necessitating the identification of novel tumor markers [45]. LncRNAs are transcribed by RNA polymerase II, and their expression levels vary significantly between tumors and the corresponding normal tissues. Studies show that lncRNAs regulate gene expression through X chromosome silencing, chromatin modification, transcriptional interference, and activation, which in turn regulate various physiological and pathological processes [46]. The lncRNA *BCYRN1* is upregulated in multiple cancers and is therefore a potential diagnostic biomarker and therapeutic target. In addition, aberrant *BCYRN1* expression is also related to the neurodegeneration underlying Alzheimer's disease [47]. We conducted a meta-analysis of 12 studies including 1,284 cancer patients and 10 distinct cancer types and found that *BCYRN1* overexpression in the tumors correlated significantly to poor survival, worse clinical stage, distant tumor metastasis, and advanced tumor T stage with greater invasiveness. Our findings are in line with previous reports indicating the prognostic relevance of *BCYRN1* in cancer. Finally, we further evaluated the prognostic and pathological value of *BCYRN1* by downloading relevant data using public databases, and the results were consistent with our meta-analysis.

Three studies included in the meta-analysis reported increased expression of *BCYRN1* in lung cancers, which correlated with poor outcomes. Wang et al. showed that *BCYRN1* promoted the proliferation and metastasis of NSCLC cells by activating the Wnt/β-catenin signaling pathway [15]. Another study reported an association between *BCYRN1* and advanced tumor stage and metastasis in NSCLC patients. *BCYRN1* augmented the malignant development by targeting H1299/DDP-induced apoptosis [16]. Furthermore, Hu and Lu found that c-myc-activated *BCYRN1* controlled NSCLC cell metastasis by upregulating MMP9 and MMP13 [17]. Three studies analyzed the relationship between *BCYRN1* and liver cancer and reported upregulation of *BCYRN1* in the tumor tissues. Ding et al. identified the *BCYRN1*/miR-490-3p/POU3F2 ceRNA regulatory network mediating reduced survival and increased tumor cell proliferation and metastasis in HCC patients [18]. Tan et al. found that *BCYRN1* influences hepatoma cell proliferation and migration by modulating the expression of the c-Myc protein [19], and Ming et al. showed that *BCYRN1* regulates tumor-associated pathways and promotes hepatocarcinogenesis via lncRNA-miRNA-mRNA networks [20]. The upregulation of *BCYRN1* in colorectal cancer was reported in four studies. Wu et al. found that knocking down BC200 decreased invasion and epithelial-mesenchymal transition (EMT) in HCT-116 and HT29 cells via the downregulation of MMP-2 and MMP-9 [21]. Yang et al. showed that *BCYRN1* functioned as an oncogene in colorectal cancer via the miR-204-3p/KRAS axis [22]. In addition, Yu and Chen reported that the aberrantly high expression of *BCYRN1* in colorectal cancer tissues increased metastasis and worsened patient prognosis [23]. Likewise, *BCYRN1* overexpression was linked to larger tumors and advanced pathological stages in colorectal cancer patients [24]. Two studies so far have analyzed the relationship between *BCYRN1* expression and prostate cancer. Zheng et al. showed that the high expression of *BCYRN1* in prostate cancer tissues induced BCA lymphatic metastasis by activating VEGF-C/VEGFR3 signaling [25]. Huo et al. found that *BCYRN1* enhanced HDAC11 levels and promoted prostate cancer cell proliferation, glucose metabolism, and survival by targeting miR-939-3p [26]. Two studies showed a link between *BCYRN1* expression and gliomas. Mu et al. showed that *BCYRN1* is downregulated in gliomas and controls CUEDC2 expression and the PTEN/AKT/p21 pathway to suppress tumor progression by competitively binding to miR-619-5p [48]. Su et al. on the other hand reported overexpression of *BCYRN1* in gliomas and found that it targets the BC200/miR218-5p signaling axis to overcome T mozolamide resistance and inhibit tumor growth [37]. The link between *BCYRN1* expression and ESCC has been reported in two studies. Zhao et al. showed that BC200 enhances esophageal cancer cell metastasis and controls the expression of ATF4 and its downstream genes [27] and that patients with high BC200 expression exhibited worse disease-free and overall survival [28]. There are two reports investigating the link between *BCYRN1* expression and stomach cancer. Zhai and Li found that *BCYRN1* is highly expressed in gastric cancer tissues and controls gastric cancer cell proliferation, cell cycle, migration, and invasion by targeting miR-204-5p [29]. Ren et al. reported similar findings [30]. Peng et al. discovered that *BCYRN1* was highly expressed in cervical cancer and that miR-138 inhibition increased cervical cancer proliferation and invasion [31]. In addition, *BCYRN1* is upregulated in extranodal lymphomas and may enhance ASP resistance by activating autophagy [36]. BC200 is expressed at low levels in ovarian cancer and may inhibit tumor cell proliferation [32]. Singh et al. found that BC200 is upregulated in breast cancer and is a potential target for estrogen-dependent breast cancer [33]. Except for gliomas and ovarian cancer, *BCYRN1* is highly expressed in most malignancies, and the oncogenic mechanisms need future investigation. The mechanism and research progress of *BCYRN1* in various types of cancer are shown in Table 3.

There are several limitations to our study that ought to be considered. Since all studies had been conducted in China, our findings may only apply to Asian patients. Second, only a tiny percentage of cases were included, and several cancer types had limited sample sizes. To overcome these restrictions, we analyzed the gene in an existing public database to validate and increase the reliability of the results. Third, manually deriving HRs for OS and PFS from Kaplan–Meier curves might lead to operational mistakes. Fourth, we did not have a uniform threshold value for high and low *BCYRN1* expression, since some studies used median and others used mean. Fifth, this study used meta-analysis and bioinformatics analysis to make a preliminary summary and judgment of the prognosis and expression of *BCYRN1* in tumors, providing a theoretical basis for future research in this area, but the lack of specific laboratory validation is a great regret. Sixth, this paper is an analysis of pan-cancer, but there is heterogeneity between each cancer, and *BCYRN1* can be specifically analyzed in separate cancer types in the future. Finally, all studies were published in English, which may have led to selection bias.

## 5. Conclusion

Elevated *BCYRN1* expression is correlated to worse prognosis and clinicopathological features (including T stage, clinical stage, and distant tumor metastasis) in cancer patients. There was no significant relationship between high *BCYRN1* expression and patient age, gender, tumor differentiation, lymphatic metastasis, or tumor size. Thus, *BCYRN1* is a potential diagnostic biomarker and therapeutic target in various cancers, although the underlying mechanisms and clinical significance have to be corroborated further with large-scale, multicenter cohort studies.

## Figures and Tables

**Table 1:** Characteristics of the included studies.

Author	Year	Country	Cancer type	Sample size (high/low)	Sample	Survival analysis	Detection method	Cutoff value	Extract method of HR	Follow-up time	NOS score
Zheng et al. [25]	2021	China	BLC	210 (105/105)	Tissue	OS DFS	qRT-PCR	Median	Data in paper	96 months	8
Wang et al. [36]	2021	China	ENKTCL	40 (20/20)	Tissue	PFS	qRT-PCR	Median	Survival curves	40 months	7
Su et al. [37]	2020	China Taiwan	GB	48 (25/23)	Tissue	NR	qRT-PCR	NR	NR	NR	6
Huo et al. [26]	2020	China	PRCA	72 (36/36)	Tissue	NR	qRT-PCR	NR	NR	NR	7
Zhai and Li [29]	2019	China	GC	127 (63/64)	Tissue	OS	qRT-PCR	Median	Data in paper	90 months	8
Yu and Chen [23]	2019	China	CRC	150 (79/71)	Tissue	OS	qRT-PCR	Mean	Survival curves	60 months	8
Ming et al. [20]	2019	China	HCC	73 (37/36)	Tissue	OS	qRT-PCR	Median	Survival curves	50 months	7
Gao and Wang [16]	2019	China	NSLC	76 (32/44)	Tissue	OS	qRT-PCR	Mean	Survival curves	70 months	7
Lin [38]	2018	China	HCC	240	Tissue	OS	qRT-PCR	Mean	Survival curves	80 months	8
Wu et al. [21]	2018	China	CC	82	Tissue	OS	qRT-PCR	Mean	Survival curves	80 months	7
Gu et al. [24]	2018	China	CRC	96 (63/33)	Tissue	NR	qRT-PCR	NR	NR	NR	6
Zhao et al. [28]	2016	China	ESCC	70 (35/35)	Tissue	OS DFS	qRT-PCR	Median	Data in paper	50 months	8

*Note.* BLC: bladder cancer, ENKTCL: extranodal NK/T-cell lymphoma, GB: glioblastoma, PRCA: prostate cancer, GC: gastric cancer, CRC: colorectal cancer, HCC: hepatocellular carcinoma, NSLC: non-small-cell lung cancer, CC: colon cancer, ESCC: esophageal squamous cell carcinoma, qRT-PCR: quantitative real-time polymerase chain reaction, and NR: not reported.

**Table 2:** Association of *BCYRN1* expression with clinicopathological features and survival prognosis.

Outcome	Studies (*n*)	OR/HR	95% CI	*p* value	Model	Heterogeneity		
						Chi^2^	*I* ^2^	*p* value
Age (≥60 vs.<60)	8	1.12	0.82–1.15	0.475	Fixed	6.09	0.0%	0.529
Gender (male vs. female)	7	0.89	0.63–1.24	0.568	Fixed	4.81	0.0%	0.568
Tumor size (large vs. small)	2	1.61	0.82–3.15	0.166	Fixed	1.64	39%	0.200
Lymph node metastasis (positive vs. negative)	4	2.09	0.79–5.51	0.135	Random	14.55	79.4	0.002
Tumor differentiation (bad vs. well)	5	1.10	0.59–2.05	0.774	Random	10.61	62.3%	0.031
TNM stage (III-IV vs. I-II)	4	2.52	1.18–5.37	0.017	Random	9.79	67.3%	0.020
Tumor T stage (III-IV vs. I-II)	6	2.11	1.49–2.99	0.000	Fixed	4.94	0.0%	0.423
Distant metastasis (positive vs. negative)	4	4.19	1.45–12.05	0.008	Random	8.19	63.4%	0.042
Overall survival (OS)	8	1.84	1.51–2.25	0.000	Fixed	1.91	0.0%	0.964
Disease-free survival (DFS)	2	1.65	1.20–2.26	0.002	Fixed	0.86	0.0%	0.353

**Table 3:** Summary of lncRNA *BCYRN1* functional roles and related genes.

Cancer types	Expression	Potential targets	Pathways and mechanisms	Related microRNAs	References
NSCLC	Up	*β*-Catenin/c-Myc/cyclin D1	Cell proliferation and migration↑; Wnt/*β*-catenin signaling	NR	[15]
		PI3K/AKT/STAT3	Cell proliferation, invasion, and migration↑; PI3K/AKT pathway	NR	[16]
		c-MYC	Cell metastasis↑; promoting the expressions of MMP9 and MMP13	NR	[17]
HCC	Up	POU3F2	Cells growth, clone formation, and movement abilities↑	miR-490-3p	[18]
		c-MYC/Bcl-xL	Affected the proliferation and migration of HepG2 cells; reduced the expression of Bcl-xL protein	NR	[19]
Colon cancer	Up	STAT3/*β*-catenin	Proliferation↑; apoptosis↓; reduction of the phosphorylation of STAT3	NR	[21]
CRC.	Up	KRAS	Proliferation, migration, and invasion ↑; apoptosis↓	miR-204-3p	[22]
		CCA T2	CCA T2; miR-320a axis	miR-320a	[23]
		NPR3	proliferation↑; apoptosis↓	NR	[24]
BLC	UP	WNT5A/VEGF-C/VEGFR3	Activates WNT5A/vegf-c/vegfr3 feedforward loop to drive lymphatic metastasis	NR	[25]
	Up	HDAC11	Sponged miR939-3p to upregulate histone deacetylase 11 (HDAC11) expression	miR-939-3p	[26]
Glioma	Down	CUEDC2	Regulate CUEDC2 expression and the PTEN/akt/p21 pathway	miR-619-5p	[48]
ESCC	Up	ATF4	Cell invasion and migration↑	NR	[27]
GC	Up	NR	Cell proliferation, cell cycle, migration, and invasion↑	miR-204-5p	[29]
		EpCAM	Cell proliferation and metastasis↑; apoptosis↓	NR	[30]
Cervical cancer	Up	NR	The proliferation and invasion ↑of cervical cancer via targeting miR-138	miR-138	[31]
ENKTCL	Up	PI3K/AKT/mTOR/p53	PI3K/AKT/mTOR/p53 pathways	NR	[36]
Breast cancer	Up	Bcl-xL	Apoptosis↓	NR	[33]

*Note.* NSCLC: non-small-cell lung cancer, HCC: hepatocellular carcinoma, CRC: colorectal cancer, BLC: bladder cancer, ESCC: esophageal squamous cell carcinoma, ENKTCL: extranodal NK/T-cell lymphoma, NR: not reported, ↑: promote, and ↓: inhibit.

## Data Availability

The analyzed data sets generated during the study are available from the corresponding author upon reasonable request.

## Abbreviation

ACC: adrenocortical carcinoma
BCYRN1: brain cytoplasmic RNA 1
BLCA: Bladder urothelial carcinoma
BRCA: Breast invasive carcinoma
CESC: Cervical squamous cell carcinoma and endocervical adenocarcinoma
CHOL: Cholangio carcinoma
CI: Confidence interval
COAD: Colon adenocarcinoma
DFI: Disease-free interval
DFS: Disease-free survival
DLBC: Lymphoid neoplasm diffuse large B-cell lymphoma
DSS: Disease-specific survival
ESCA: Esophageal carcinoma
GBM: Glioblastoma multiforme
GEPIA: Gene expression profiling interactive analysis
GTEx: The genotype-tissue expression
HCC: Hepatocellular carcinoma
HNSC: Head and neck squamous cell carcinoma
HR: Hazard ratio
KICH: Kidney chromophobe
KIRC: Kidney renal clear cell carcinoma
KIRP: Kidney renal papillary cell carcinoma
LAML: Acute myeloid leukemia
LGG: Brain lower-grade glioma
LIHC: Liver hepatocellular carcinoma
LncRNA: Long non-coding RNA
LNM: Lymph node metastasis
LUAD: Lung adenocarcinoma
LUSC: Lung squamous cell carcinoma
MESO: Mesothelioma
miRNA: MicroRNA
mRNA: Messenger RNA
MSI: Microsatellite instability
NOS: Newcastle–Ottawa scale
NSCLC: Non-small-cell lung cancer
OR: Odds ratio
OS: Overall survival
OV: Ovarian serous cystadenocarcinoma
PAAD: Pancreatic adenocarcinoma
PCPG: Pheochromocytoma and paraganglioma
PFI: Progression-free interval
PRAD: Prostate adenocarcinoma
READ: Rectum adenocarcinoma
RFS: Recurrence-free survival
SARC: Sarcoma
SKCM: Skin cutaneous melanoma
STAD: Stomach adenocarcinoma
TCGA: The cancer genome atlas
TGCT: Testicular germ cell tumors
THCA: Thyroid carcinoma
THYM: Thymoma
TMB: Tumor mutational burden
TME: Tumor immune microenvironment
UCEC: Uterine corpus endometrial carcinoma
UCS: Uterine carcinosarcoma
UVM: Uveal melanoma.
